# Association between iron deficiency anemia and risk of hearing loss among female patients: a multi-institutional cohort study

**DOI:** 10.3389/fnut.2025.1718261

**Published:** 2025-12-10

**Authors:** I-Wen Chen, Ting-Sian Yu, Yi-Chen Lai, Ying-Jen Chang, Kuo-Chuan Hung

**Affiliations:** 1 Department of Anesthesiology, Chi Mei Medical Center, Liouying, Tainan, Taiwan; 2 Department of Anesthesiology, E-Da Hospital, I-Shou University, Kaohsiung, Taiwan; 3 Department of Anesthesiology, Chi Mei Medical Center, Tainan, Taiwan

**Keywords:** iron deficiency anemia, hearing loss, nutrition, cohort study, hazard ratio

## Abstract

**Background:**

Iron deficiency anemia (IDA) represents the most prevalent nutritional deficiency worldwide, disproportionately affecting women of reproductive age. Emerging evidence suggests IDA may have underappreciated consequences for sensory organ function, particularly hearing. The inner ear’s high metabolic demands and intricate vascular supply may render it vulnerable to chronic IDA and resulting tissue hypoxia. This study examined the association between IDA and hearing loss risk in female patients.

**Methods:**

This retrospective cohort study used the TriNetX Analytics Network Platform database (2010–2022) with five-year follow-up. Female patients with IDA were identified by hemoglobin below 12 g/dL, serum ferritin below 30 ng/mL within 3 months, and ICD-10-CM diagnosis codes. Controls had hemoglobin above 12 g/dL, ferritin above 30 ng/mL, and no IDA codes. Propensity score matching created 71,003 matched pairs balanced for demographics, comorbidities, and laboratory parameters. The primary outcome was hearing loss development within 5 years. Time-to-event analyses used Kaplan–Meier methods and Cox proportional hazards regression.

**Results:**

Patients with IDA demonstrated significantly higher hearing loss incidence compared to matched controls (0.98% versus 0.81%, hazard ratio [HR]: 1.50, 95% CI 1.34–1.67, *p* < 0.001). Risk was highest within the first year (HR 2.79, 95% CI 2.00–3.88) and remained significant at 5 years. Dose–response analysis revealed greater risk with severe anemia (hemoglobin below 10 g/dL). Age-stratified analysis showed consistent associations across age groups, and findings were replicated in male patients.

**Conclusion:**

IDA is independently associated with increased hearing loss risk in women, with a temporal gradient suggesting acute effects during active deficiency. These findings suggest routine audiological assessment should be considered for patients with IDA, particularly during the first year following diagnosis.

## 1 Introduction

Iron deficiency anemia (IDA) represents the most prevalent nutritional deficiency worldwide, with a disproportionate burden on women of reproductive age (1–3). It develops when the body’s iron reserves are exhausted, resulting in diminished hemoglobin production and a subsequent reduction in oxygen-carrying capacity (4, 5). Beyond its well-recognized systemic effects including fatigue, reduced exercise tolerance, and cognitive impairment (6–8), emerging evidence suggests that IDA may have previously underappreciated consequences for sensory organ function, particularly hearing (9–13). The inner ear, with its high metabolic demands and intricate vascular supply, may be particularly vulnerable to the effects of chronic IDA and the resulting tissue hypoxia (14–16).

Although several studies have linked IDA to hearing loss, the overall evidence remains limited and insufficiently explored. For instance, the study by Schieffer et al. (9) included only 2,274 patients with IDA and failed to implement propensity score matching or adequate control for patient characteristics, potentially introducing a significant confounding bias. Mohammed et al. (17) conducted a systematic review and meta-analysis of four studies, finding that individuals with IDA had 55% higher odds of developing sensorineural hearing loss compared to those without anemia. However, most available studies in the meta-analysis (17) are cross-sectional, lacking the temporal resolution and statistical power to establish causality or evaluate risk progression over time. Critically, existing research has not specifically focused on female populations, despite women representing the highest-risk demographic for IDA due to menstruation, pregnancy, and dietary factors. This gap is particularly problematic because studying mixed-sex populations introduces substantial confounding from occupational noise exposure, which disproportionately affects males (18) and obscures the independent relationship between nutritional deficiencies and auditory function.

Therefore, we conducted this large-scale, multi-institutional cohort study to examine the association between IDA and the risk of hearing loss, specifically in female patients. By focusing exclusively on women, we aimed to minimize confounding from occupational noise exposure while leveraging a population with a higher baseline prevalence of IDA.

## 2 Methods

### 2.1 Study design and data source

We conducted a retrospective cohort study using the TriNetX Analytics Network Platform, a large-scale, multi-institutional database that aggregates de-identified electronic health records from participating healthcare organizations across multiple countries. The database encompasses comprehensive clinical variables, including demographics, diagnoses recorded using the International Classification of Diseases codes, laboratory measurements, medication prescriptions, and procedural records. The Institutional Review Board of Chi Mei Medical Center approved this study (IRB number: 11403-E01) and waived the requirement for informed consent, given the retrospective nature of the analysis and the use of de-identified data.

### 2.2 Study population

We identified female patients with and without IDA between January 1, 2010, and December 31, 2022, from the TriNetX database. Our study focused on female patients to minimize confounding from occupational noise exposure, which disproportionately affects males (18) and could not be adequately controlled in our database.

For the IDA group, we applied several diagnostic criteria to maximize case identification accuracy. Patients were classified as having IDA if they met all of the following conditions (index date): (1) a hemoglobin level below 12 g/dL; (2) a serum ferritin concentration below 30 ng/mL recorded within 3 months of the hemoglobin test; and (3) at least one ICD-10-CM diagnosis code for IDA during the follow-up period. To ensure data integrity, we further excluded patients with serum ferritin levels exceeding 30 ng/mL at any point during the observation period. This hemoglobin threshold was consistent with the diagnostic criteria recommended by the World Health Organization (19), which defines anemia in non-pregnant women as hemoglobin <12 g/dL.

The control group included female patients without IDA, defined as hemoglobin levels above 12 g/dL and serum ferritin concentrations exceeding 30 ng/mL within the same three-month window. Patients were excluded if they had any ICD-10-CM diagnosis codes for IDA or serum ferritin levels below 30 ng/mL during the entire follow-up period. To address the potential bias from differential healthcare utilization, where healthier controls might have fewer medical encounters and thus fewer opportunities for diagnosis, we required all control patients to have at least three hemoglobin measurements recorded during the study period. This criterion helped ensure comparable levels of medical surveillance between the two groups.

### 2.3 Exclusion criteria

To ensure that we focused on new-onset hearing loss and reduce potential bias, we systematically excluded patients with any prior diagnosis of sickle cell disorders (ICD-10-CM D57) (20), conductive or sensorineural hearing loss (ICD-10-CM H90), unspecified hearing loss (ICD-10-CM H91), surgical procedures involving the inner ear (CPT 1010223) or middle ear (CPT 1010147), and neoplasms (ICD-10-CM C00–D49). Additionally, we excluded patients in both groups who developed other forms of anemia during follow-up, including nutritional anemia, hemolytic anemia, aplastic anemia, and unspecified anemia. This approach ensured a clear distinction between IDA and other hematologic disorders with potentially different pathophysiological relationships with hearing loss.

### 2.4 Propensity score matching strategy

To address confounding by indication and to create comparable groups, we implemented propensity score matching. Baseline characteristics were extracted from the three-year period preceding the index date to capture comprehensive patient profiles. These variables included demographic factors such as age and race, body mass index, and comorbidities (e.g., hypertension, heart failure, ischemic heart disease, and diabetes). Laboratory parameters incorporated into the matching algorithm included hemoglobin A1c values, serum albumin concentrations, and estimated glomerular filtration rate. In addition, lifestyle-related covariates available in the TriNetX database, including nicotine dependence and alcohol-related disorders, were incorporated into the matching algorithm. These variables served as proxies for smoking and alcohol consumption behaviors, respectively. The matching employed a greedy nearest-neighbor algorithm without replacement, using a caliper width of 0.1 standard deviations of the logit of the propensity score.

### 2.5 Study outcomes

The primary outcome was the development of overall hearing loss within 5 years of the index date, encompassing both conductive/sensorineural hearing loss (ICD-10 code H90) and unspecified hearing loss (ICD-10 code H91). To provide insights into the temporal relationship between IDA and hearing loss, we evaluated outcomes at multiple time points: 1 year, 3 years, and 5 years post-index date. Secondary analyses examined specific hearing loss subtypes (i.e., conductive/sensorineural hearing loss and unspecified hearing loss) separately to determine whether IDA differentially affected various pathophysiological mechanisms of hearing impairment. To minimize potential reverse causation, such as pre-existing or undiagnosed hearing loss prompting laboratory evaluation for anemia, follow-up for outcome assessment began 3 months after the index date, and any events occurring within this period were excluded.

### 2.6 Subgroup analyses and dose–response analysis

For subgroup analysis, we stratified patients by age (18–50 years versus over 50 years) to examine whether the association varied between younger and older adults. In addition, subgroup analyses stratified by comorbidity level were performed to examine whether baseline disease burden (i.e., diabetes mellitus, chronic kidney disease, and cardiovascular disorders) modified the association between IDA and hearing loss. To investigate whether the severity of IDA influenced the risk of hearing loss, we conducted a dose–response analysis focusing on patients with severe anemia. This analysis maintained the same inclusion criteria and analytical approach as the primary analysis, except that the IDA group was restricted to female patients with hemoglobin levels below 10 g/dL.

### 2.7 Additional analysis in male patients

Although our primary analysis was limited to female patients, we also conducted a parallel analysis in male patients to evaluate the generalizability of our findings across the sexes. The analytic approach was identical to that of the primary analysis, with the application of sex-specific hemoglobin thresholds: males with hemoglobin levels below 13 g/dL were assigned to the IDA group, while those with levels above 13 g/dL constituted the control group, in accordance with the standard diagnostic criteria for anemia in males.

### 2.8 Statistical analysis

Laboratory missingness was handled according to the structure of the TriNetX platform, which does not perform data imputation. Patients without available hemoglobin or ferritin values were excluded from cohort selection. Because TriNetX provides aggregated, de-identified data from multiple healthcare organizations, individual-level records, site identifiers, and data-quality metrics are not accessible to investigators, and thus the mechanism of missingness (random or systematic) cannot be directly assessed. In addition, mixed-effects modeling or stratification by site could not be performed. However, the inclusion of a large number of healthcare organizations across diverse regions likely enhances generalizability and reduces the influence of data irregularities from any single site.

Baseline characteristics were described using means and standard deviations for continuous variables, and frequencies with percentages for categorical variables. To confirm that matching created comparable groups, we evaluated balance with standardized mean differences, considering values under 0.1 as acceptable. Event timing and cumulative incidence were explored with Kaplan–Meier estimates, and group differences were assessed using log-rank tests. Hazard ratios (HRs) with 95% confidence intervals (CIs) were derived from Cox proportional hazards models, and the proportional hazards assumption was checked using Schoenfeld residuals. Patients were followed until the first occurrence of hearing loss, death, or the end of the 5-year observation window, whichever came first. Patients could have less than 5 years of observable follow-up due to administrative censoring at the end of the study period or due to death. These forms of censoring were handled appropriately within the Kaplan–Meier and Cox proportional hazards models, which account for differential follow-up time without treating censored individuals as having experienced the event. All analyses adhered to an intention-to-treat approach, retaining participants in their original groups regardless of later changes in iron status. Statistical significance was defined as a two-sided *p* value below 0.05. All analyses were conducted within the TriNetX platform using its built-in analytical functions. Because TriNetX provides only aggregated, de-identified data, individual-level datasets are not accessible for external statistical modeling. Consequently, advanced causal inference techniques such as varying the matching caliper, inverse probability weighting, marginal structural modeling, or continuous exposure modeling using raw hemoglobin and ferritin values could not be performed.

## 3 Result

### 3.1 Patient selection and baseline characteristics

**Figure 1:**
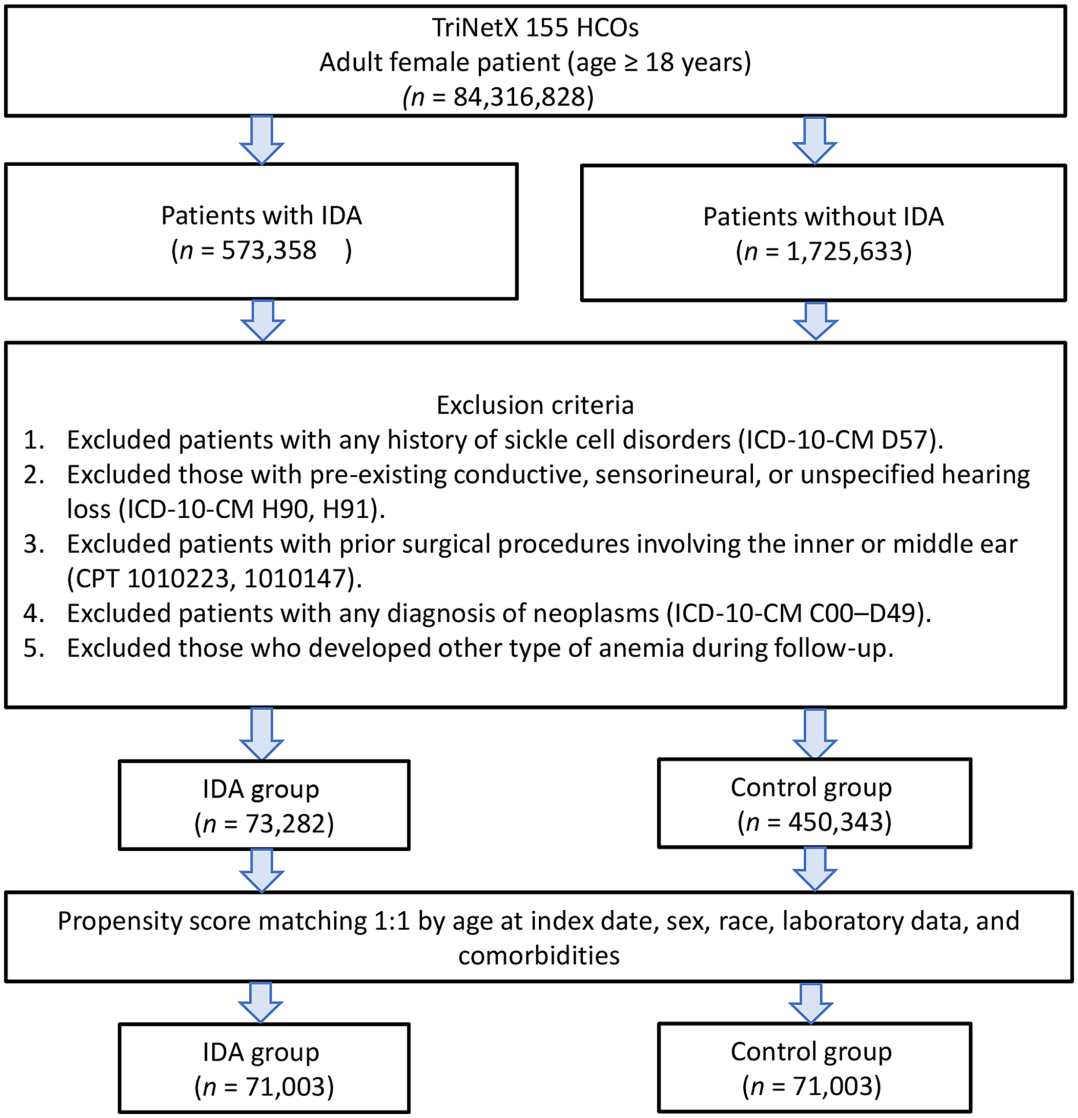
Patient selection flowchart from the TriNetX database. The flowchart illustrates the exclusion process applied to identify eligible patients with or without iron deficiency anemia (IDA). HCOs, Healthcare Organization; ICD-10-CM, International Classification of Diseases, Tenth Revision, Clinical Modification; CPT, Current Procedural Terminology.

From the TriNetX database, we identified 73,282 female patients with IDA and 450,343 controls without IDA between 2010 and 2022. After propensity score matching, the final analysis included 71,003 patients in each group (Figure 1). The matching process successfully balanced baseline characteristics between groups, as evidenced by standardized mean differences below 0.1 for all variables (Table 1). Before matching, substantial differences existed between groups, particularly in age (38.8 vs. 50.7 years) and racial distribution. After matching, both groups had comparable mean ages (39.3 vs. 39.4 years) and similar distributions across all demographic and clinical variables. The matched cohorts showed a balanced prevalence of major comorbidities, including hypertension (15.6% vs. 16.4%), diabetes mellitus (8.0% vs. 8.2%), and chronic kidney disease (1.8% vs. 2.0%).

**Table 1:** Baseline characteristics of patients before and after propensity score matching.

Variables	Before matching			After matching		
	IDA group (*n* = 73,282)	Control group (*n* = 450,343)	SMD†	IDA group (*n* = 71,003)	Control group (*n* = 71,003)	SMD†
Patient characteristics						
Age at index (years)	38.8 ± 17.6	50.7 ± 19.5	0.639	39.3 ± 17.6	39.4 ± 17.9	0.005
BMI > 30 kg/m2	17,902 (24.4%)	105,241 (23.4%)	0.025	17,441 (24.6%)	18,473 (26.0%)	0.033
White	31,897 (43.5%)	244,588 (54.3%)	0.217	31,897 (44.9%)	32,147 (45.3%)	0.007
Black or African American	18,414 (25.1%)	45,123 (10.0%)	0.405	16,366 (23.1%)	15,983 (22.5%)	0.013
Unknown Race	13,247 (18.1%)	126,047 (28.0%)	0.237	13,246 (18.7%)	12,771 (18.0%)	0.017
Other Race	5,040 (6.9%)	17,728 (3.9%)	0.130	4,913 (6.9%)	5,013 (7.1%)	0.006
Asian	3,750 (5.1%)	14,194 (3.2%)	0.099	3,695 (5.2%)	4,176 (5.9%)	0.030
Factors influencing health status and contact with health services	41,186 (56.2%)	202,260 (44.9%)	0.227	39,317 (55.4%)	41,193 (58.0%)	0.053
Comorbidities						
Essential (primary) hypertension	11,259 (15.4%)	83,861 (18.6%)	0.087	11,101 (15.6%)	11,665 (16.4%)	0.022
Overweight and obesity	10,826 (14.8%)	61,105 (13.6%)	0.035	10,513 (14.8%)	11,075 (15.6%)	0.022
Dyslipidemia	7,484 (10.2%)	66,754 (14.8%)	0.140	7,430 (10.5%)	7,643 (10.8%)	0.010
Diabetes mellitus	5,771 (7.9%)	39,357 (8.7%)	0.031	5,702 (8.0%)	5,846 (8.2%)	0.007
Sleep disorders	4,967 (6.8%)	38,008 (8.4%)	0.063	4,920 (6.9%)	4,950 (7.0%)	0.002
Nicotine dependence	4,004 (5.5%)	22,878 (5.1%)	0.017	3,918 (5.5%)	3,819 (5.4%)	0.006
Diseases of liver	1759 (2.4%)	23,009 (5.1%)	0.143	1759 (2.5%)	1870 (2.6%)	0.010
Cerebrovascular diseases	1725 (2.4%)	13,927 (3.1%)	0.045	1708 (2.4%)	1,684 (2.4%)	0.002
COVID-19	1,579 (2.2%)	24,591 (5.5%)	0.173	1,579 (2.2%)	1829 (2.6%)	0.023
Chronic kidney disease (CKD)	1,299 (1.8%)	15,994 (3.6%)	0.111	1,297 (1.8%)	1,429 (2.0%)	0.014
Systemic connective tissue disorders	1,047 (1.4%)	8,646 (1.9%)	0.038	1,043 (1.5%)	1,090 (1.5%)	0.005
Alcohol related disorders	959 (1.3%)	6,583 (1.5%)	0.013	949 (1.3%)	923 (1.3%)	0.003
Malnutrition	692 (0.9%)	5,245 (1.2%)	0.022	684 (1.0%)	671 (0.9%)	0.002
Laboratory data						
Hemoglobin A1c ≥ 9%	2,680 (3.7%)	24,850 (5.5%)	0.089	2,669 (3.8%)	2,613 (3.7%)	0.004
Albumin g/dL (≥3.5 g/dL)	29,818 (40.7%)	230,649 (51.2%)	0.212	29,583 (41.7%)	30,841 (43.4%)	0.036
eGFR>60 mL/min/1.73 m^2^	33,942 (46.3%)	266,285 (59.1%)	0.259	33,712 (47.5%)	33,868 (47.7%)	0.004

IDA, iron deficiency anemia; BMI, body mass index; SMD, standardized mean differences; † SMD values <0.1 indicate adequate balance between groups; eGFR, estimated Glomerular Filtration Rate.

### 3.2 Association between IDA and hearing loss

During the 5-year follow-up period, patients with IDA demonstrated a significantly higher incidence of overall hearing loss than matched controls (0.98% vs. 0.81%, HR 1.50, 95% CI 1.34–1.67, *p* < 0.001) (Table 2). This increased risk was consistent across both hearing loss subtypes, with conductive/sensorineural hearing loss showing a 47% increased risk (HR 1.47, 95% CI 1.26–1.72) and unspecified hearing loss demonstrating a 53% increased risk (HR 1.53, 95% CI 1.34–1.74). At 5 years, the absolute risk differences between IDA and control groups were 0.17% for overall hearing loss, 0.075% for conductive/sensorineural hearing loss, and 0.13% for unspecified hearing loss (Table 2).

**Table 2:** Association between iron deficiency anemia and hearing loss at 5-year follow up.

Outcomes	IDA group (*n* = 71,003)	Control group (*n* = 71,003)	HR (95% CI)	*p*-value	*p*-value for proportionality	Risk difference
	Events (%)	Events (%)				
Overall HL	698 (0.98%)	577 (0.81%)	1.50 (1.34–1.67)	<0.001	0.100	0.17%
Conductive/sensorineural HL	344 (0.48%)	291 (0.41%)	1.47 (1.26–1.72)	<0.001	0.377	0.075%
Unspecified HL	488 (0.69%)	396 (0.56%)	1.53 (1.34–1.74)	<0.001	0.191	0.13%

IDA, iron deficiency anemia; HL, hearing loss; HR, hazard ratio; CI, confidence interval.

**Figure 2:**
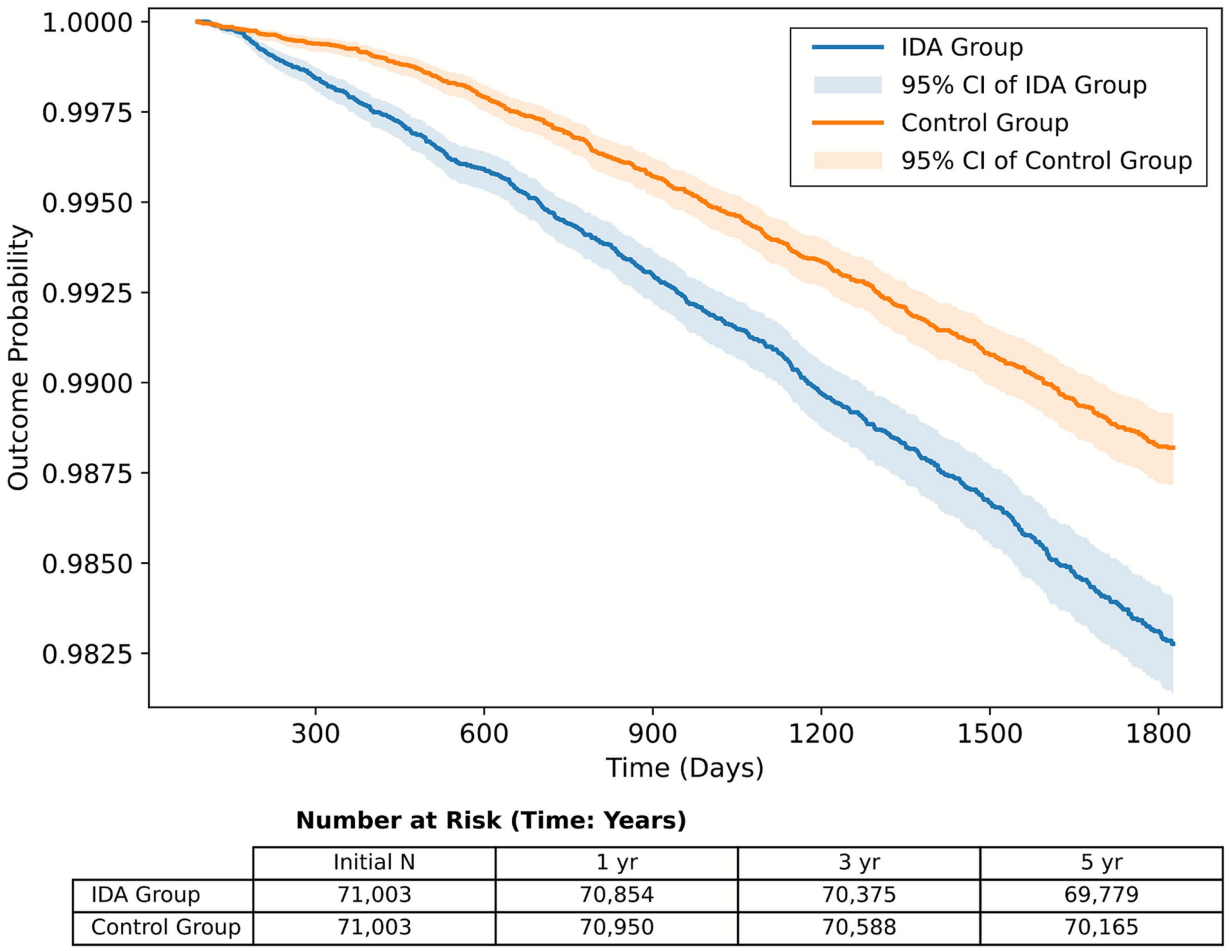
Kaplan–Meier curves for cumulative incidence of hearing loss among matched female patients with and without iron deficiency anemia (IDA). Time zero was defined as the index date when all diagnostic criteria for IDA were first met. Follow-up for outcome assessment began 3 months after the index date, and any hearing loss events occurring within this period were excluded to minimize potential reverse causation. Separate curves are shown for the IDA and control cohorts, with shaded areas representing 95% confidence intervals (CIs). The number of patients at risk at each time point is displayed below the plot.

Temporal trends demonstrated that the risk of hearing loss linked to IDA sharply increased soon after diagnosis (Table 3). Patients with IDA had nearly three times the risk of developing overall hearing loss within the first year (HR 2.79, 95% CI 2.00–3.88, *p* < 0.001), with this elevated risk gradually attenuating but remaining statistically significant at 3 years (HR 1.56, 95% CI 1.35–1.79). This temporal gradient suggests that the effect of IDA on auditory function may be most pronounced during periods of active deficiency, highlighting the potential importance of early intervention (Figure 2).

**Table 3:** Association between iron deficiency anemia and hearing loss at 1- and 3-year follow-up.

Outcomes	1-year outcomes		3-year outcomes	
	HR (95% CI)	*p*-values	HR (95% CI)	*p*-values
Overall HL	2.79 (2.00–3.88)	<0.001	1.56 (1.35–1.79)	<0.001
Conductive/sensorineural HL	2.35 (1.47–3.75)	<0.001	1.57 (1.28–1.92)	<0.001
Unspecified HL	3.14 (2.06–4.80)	<0.001	1.58 (1.33–1.87)	<0.001

IDA, iron deficiency anemia; HL, hearing loss; HR, hazard ratio; CI, confidence interval.

The proportional hazards assumption was evaluated using Schoenfeld residuals. The global test showed no significant violation at 1 year (*p* > 0.05) and 5 years (*p* > 0.05), whereas a deviation was observed at 3 years (*p* < 0.05). This isolated finding suggests minor temporal variation rather than a systematic violation of the proportional hazards assumption.

### 3.3 Dose–response relationship in moderate to severe anemia

To explore whether the severity of IDA influenced hearing loss risk, we conducted a dose–response analysis focusing on 40,453 matched pairs of patients with moderate-to-severe anemia (hemoglobin <10 g/dL). This analysis revealed a clear intensification of risk with greater anemia severity (Table 4). Women with more severe IDA showed an even stronger association with hearing loss at 1 year (HR 3.31, 95% CI 2.23–4.92) compared to the primary analysis. The 5-year risk remained substantially elevated (HR 1.76, 95% CI 1.53–2.03), demonstrating that more severe IDA confers a proportionally greater risk for auditory impairment. This dose–response relationship strengthens the biological plausibility of a causal association between IDA and hearing loss.

**Table 4:** Dose–response of IDA and hearing loss in female with moderate to severe anemia (Hb < 10) (*n* = 40,453 for each group).

Outcomes	1-year		3-year		5-year	
	HR (95% CI)	*p*-values	HR (95% CI)	*p*-values	HR (95% CI)	*p*-values
Overall HL	3.31 (2.23–4.92)	<0.001	1.89 (1.58–2.26)	<0.001	1.76 (1.53–2.03)	<0.001
Conductive/sensorineural HL	2.51 (1.46–4.32)	<0.001	2.02 (1.55–2.62)	<0.001	1.82 (1.49–2.22)	<0.001
Unspecified HL	3.95 (2.37–6.59)	<0.001	1.80 (1.45–2.22)	<0.001	1.76 (1.48–2.08)	<0.001

IDA, iron deficiency anemia; HL, hearing loss; HR, hazard ratio; CI, confidence interval.

### 3.4 Subgroup analysis based on age

Given the potential for age-related differences in both iron metabolism and hearing vulnerability, we stratified our analysis by age group (Table 5). Interestingly, the association between IDA and hearing loss was remarkably consistent across age groups. Among younger women (18–50 years), IDA was associated with a 45% increased risk of hearing loss at 5 years (HR 1.45, 95% CI 1.24–1.70), while older women (>50 years) showed a similar 40% increased risk (HR 1.40, 95% CI 1.20–1.63). The absence of a significant interaction between age and IDA effect (p for interaction >0.05 for all outcomes) suggests that IDA poses a similar relative risk for hearing loss regardless of age, although the absolute risk may differ. This finding underscores the importance of monitoring the iron status and hearing function across all age groups in women.

**Table 5:** Subgroup analysis based on age.

Outcomes	18–50 years (*n* = 45,620 for each group)		>50 years (*n* = 24,523 for each group)		P for interaction
	HR (95% CI)	*p*-values	HR (95% CI)	*p*-values	
1-year HL					
Overall HL	2.08 (1.32–3.26)	0.001	2.67 (1.72–4.14)	<0.001	0.456
Conductive/sensorineural HL	1.51 (0.81–2.82)	0.197	2.13 (1.17–3.90)	0.012	0.473
Unspecified HL	2.50 (1.41–4.42)	0.001	3.02 (1.70–5.34)	<0.001	0.666
3-year HL					
Overall HL	1.53 (1.25–1.86)	<0.001	1.53 (1.25–1.89)	<0.001	1.000
Conductive/sensorineural HL	1.43 (1.06–1.93)	0.019	1.51 (1.15–1.98)	0.003	0.794
Unspecified HL	1.54 (1.22–1.94)	<0.001	1.52 (1.19–1.95)	<0.001	0.940
5-year HL					
Overall HL	1.45 (1.24–1.70)	<0.001	1.40 (1.20–1.63)	<0.001	0.756
Conductive/sensorineural HL	1.37 (1.08–1.75)	0.01	1.38 (1.13–1.69)	0.002	0.964
Unspecified HL	1.49 (1.23–1.79)	<0.001	1.51 (1.25–1.82)	<0.001	0.922

IDA, iron deficiency anemia; HL, hearing loss; HR, hazard ratio; CI, confidence interval.

### 3.5 Additional analysis in male patients

To assess the generalizability of our findings beyond the female population, we conducted a parallel analysis of 11,993 matched pairs of male patients (Table 6). Male patients with IDA showed a comparable pattern of increased risk of hearing loss, with an 82% increased risk at 5 years (HR 1.82, 95% CI 1.43–2.32, *p* < 0.001). The consistency of the findings across both sexes, despite using sex-specific hemoglobin thresholds for defining anemia, suggests that the association between IDA and hearing loss represents a fundamental biological relationship rather than a sex-specific phenomenon.

**Table 6:** Association between iron deficiency anemia and hearing loss in male population (*n* = 11,993 for each group).

Outcomes	1-year†		3-year		5-year	
	HR (95% CI)	*p*-values	HR (95% CI)	*p*-values	HR (95% CI)	*p*-values
Overall HL	2.18 (1.08–4.38)	0.025	2.15 (1.57–2.94)	<0.001	1.82 (1.43–2.32)	<0.001
Conductive/sensorineural HL	–	–	1.86 (1.18–2.92)	0.007	1.75 (1.26–2.45)	<0.001
Unspecified HL	–	–	2.18 (1.49–3.19)	<0.001	1.60 (1.20–2.15)	0.001

IDA, iron deficiency anemia; HL, hearing loss; HR, hazard ratio; CI, confidence interval; † For the 1-year outcome, the event count was insufficient for reliable statistical estimation within the TriNetX system; therefore, detailed results are not displayed.

## 4 Discussion

In this large, multicenter cohort study, we identified a significant link between IDA and hearing loss in female patients. Among 71,003 propensity score-matched pairs followed over 5 years, women with IDA exhibited a 50% higher risk of developing hearing loss than matched controls. This association was consistently observed for both conductive/sensorineural and unspecified types of hearing loss. The risk was especially elevated within the first year after an IDA diagnosis, when the hazard ratio approached three, but it remained statistically significant throughout the entire follow-up period. Collectively, these results demonstrate a statistically significant association between IDA and auditory impairment in women; however, given the small absolute risk increase, these findings should be interpreted with appropriate caution rather than as evidence of a large population-level effect.

Several plausible biological mechanisms may explain the observed association between IDA and hearing loss. The cochlea’s unique vascular architecture makes it particularly vulnerable to ischemic damage, as it receives blood supply exclusively from the labyrinthine artery without collateral circulation (21, 22). IDA reduces the oxygen-carrying capacity of hemoglobin, potentially leading to cochlear hypoxia and subsequent damage to the highly metabolically active hair cells and stria vascularis. Additionally, iron serves as a crucial cofactor in numerous enzymatic processes that are essential for cellular metabolism, including energy production and neurotransmitter synthesis (23, 24). IDA may compromise the structural integrity of auditory nerve myelin, as iron is required for lipid synthesis and myelin maintenance (25). Furthermore, iron deficiency can lead to increased platelet aggregation and altered blood viscosity, potentially contributing to microvascular compromise within the delicate cochlear circulation (26, 27).

In the current study, the markedly elevated risk during the first year suggests that acute IDA may precipitate immediate auditory damage through acute ischemic mechanisms affecting the cochlear microvasculature. This critical window suggests that clinicians may maintain heightened awareness of auditory symptoms following IDA diagnosis, especially in patients with more severe anemia. Although the relative risk elevations were statistically significant, it is important to acknowledge that the absolute risk differences were small (0.17% at 5 years), and our findings should therefore be viewed as hypothesis-generating rather than supporting routine audiological screening for all patients with IDA. Targeted assessment may be more appropriate for patients with persistent symptoms or additional otologic risk factors. The gradual attenuation of risk over time may reflect either the natural resolution of IDA through treatment or the development of compensatory physiological mechanisms. However, the persistence of statistically significant risk throughout the entire five-year follow-up period demonstrates that the effects of IDA on auditory function may have lasting consequences. Our longitudinal analysis offers essential temporal insights that were not captured in previous cross-sectional studies. For example, a study by Schieffer et al. (9) identified a similar association between IDA and hearing loss, but lacked follow-up data to track risk progression over time. Similarly, a meta-analysis by Mohammed et al. reported a comparable pooled odds ratio (OR 1.55), yet its evidence base consisted primarily of cross-sectional studies (17). In contrast, our five-year follow-up design uncovered a critical risk window during the first year after IDA diagnosis and demonstrated that elevated risk persists over the long term. These findings directly inform decisions regarding the timing and intensity of audiological surveillance, providing a level of temporal detail that is absent from previous investigations.

The dose–response analysis of patients with moderate-to-severe anemia provides even stronger evidence for a biological gradient linking the severity of IDA to the risk of hearing loss. Women whose hemoglobin levels fell below 10 g/dL demonstrated substantially higher hazard ratios across every assessed time interval, with first-year risk increasing to 3.31 times that observed in matched controls. This consistent gradient reinforces the argument for a causal relationship, as greater exposure intensity corresponded with larger effect sizes, a key feature supporting biological plausibility. These findings indicate that a more profound IDA may inflict proportionally greater cochlear injury. From a clinical perspective, this relationship underscores the importance of heightened audiological monitoring, earlier detection strategies, and, where appropriate, more aggressive iron replacement therapy to mitigate long-term auditory complications in patients with severe anemia.

In our analysis, both younger women (18–50 years) and older women (>50 years) demonstrated comparable relative risk increases at the years of follow-up. This age independence suggests that IDA exerts its effects on auditory function through fundamental physiological processes, such as cochlear oxygen transport and neurotransmitter metabolism, rather than through pathways unique to specific life stages. Importantly, the absence of a significant interaction between age and the effect of IDA indicates that the role of iron in maintaining cochlear integrity remains critical throughout the adult lifespan. These findings suggest that screening for and addressing IDA should be considered equally essential for hearing preservation across all adult age categories, not just among elderly individuals who traditionally receive the most attention in hearing loss prevention programs. By highlighting the broad relevance of iron homeostasis in auditory health, our results underscore the value of integrating iron status evaluation into routine care for adult women of all ages, potentially improving early detection and intervention strategies to mitigate long-term auditory complications. As the proportion of patients with major comorbidities was relatively low in this cohort, likely reflecting a generally healthier population undergoing routine hemoglobin testing, subgroup analyses by comorbidity level could not be meaningfully performed. Future studies including patients with greater comorbidity burden are needed to explore potential effect modification by multimorbidity.

Male patients with IDA demonstrated a comparable increased risk of hearing loss (HR 1.82 at 5 years) in the current study. This consistency across both sexes strongly suggests that the observed association represents a fundamental biological relationship rather than a sex-specific phenomenon or an artifact of gender-related confounding factors. The similar effect magnitude in males, despite their lower baseline IDA prevalence and different risk factor profiles, reinforces the robustness of the association between IDA and hearing loss and supports the generalizability of our findings beyond the female population.

In current study, a potential immortal-time bias cannot be completely excluded. Because the IDA group required fulfillment of all diagnostic criteria (hemoglobin <12 g/dL, ferritin <30 ng/mL within 3 months, and a corresponding ICD-10-CM code), the timing of laboratory tests and diagnostic coding may not have been perfectly aligned. In contrast, control patients with normal hemoglobin and ferritin levels might not have undergone concurrent testing, as their labs were not necessarily obtained for anemia evaluation. These differences could introduce a brief period of immortal time before IDA confirmation, although the impact is likely minimal given that hemoglobin and ferritin are often measured together in clinical practice. Although the Schoenfeld residuals test indicated a deviation from proportionality at the 3-year follow-up, the 1-year and 5-year tests showed no violation. This minor fluctuation likely reflects transient variation in risk rather than a fundamental model violation.

Several limitations should be acknowledged when interpreting these findings. First, the observational study design precludes definitive causal inferences, and unmeasured confounding factors may have contributed to the observed associations. Second, the reliance on ICD-10 diagnostic codes for hearing loss identification may result in under-ascertainment of mild cases or overrepresentation of clinically significant cases, potentially affecting the generalizability of our prevalence estimates. Third, the focus of this study on patients within healthcare systems may introduce selection bias, as healthier individuals with less frequent medical encounters may be underrepresented. Additionally, we lacked detailed information on iron supplementation therapy, dietary factors, or the specific causes of IDA, limiting our ability to assess whether treatment modifications might alter hearing outcomes. Additionally, baseline hearing test data were extremely limited, as audiometric results are not routinely recorded across participating institutions in the TriNetX network. Consequently, subgroup analyses based on baseline hearing status could not be meaningfully performed. Fourth, although our propensity score matching adjusted for numerous clinical and lifestyle variables, including nicotine dependence and alcohol-related disorders as proxies for smoking and alcohol use, other potentially important confounders such as income level and pregnancy status were not uniformly available across participating healthcare organizations in TriNetX. The absence of these socioeconomic and reproductive variables may contribute to residual confounding, which should be considered when interpreting the findings. Finally, as with all multi-institutional real-world databases, the TriNetX platform may be subject to variability in data completeness and coding practices across institutions. Moreover, TriNetX does not provide access to individual-level data or the detailed imputation procedures used by participating sites, as discussed in recent methodological reviews (28). Continuous exposure modeling using raw hemoglobin or ferritin values could not be performed because individual-level data are not accessible within the TriNetX platform, which only provides aggregated results.

## 5 Conclusion

Our results provide evidence that IDA is independently associated with increased hearing loss risk in both female and male adults. The dose–response relationship, temporal pattern of risk, and consistency across age subgroups support a biologically plausible association with potential clinical significance. While IDA was associated with an increased relative risk of hearing loss, the absolute risk increase was small. Therefore, these findings should be considered hypothesis-generating. Future prospective studies examining whether iron supplementation can prevent or ameliorate hearing loss, as well as investigations of optimal screening strategies and treatment protocols, are warranted to translate these epidemiological findings into clinical practice improvements.

## Data Availability

The raw data supporting the conclusions of this article will be made available by the authors, without undue reservation.
